# Complex Networks in Bioactive Peptide Research: A Methodological Review

**DOI:** 10.3390/biom16071007

**Published:** 2026-07-10

**Authors:** Kevin Castillo-Mendieta, Guillermin Agüero-Chapin, Edgar A. Márquez Brazón, José R. Mora, Noel Pérez-Pérez, Néstor Cubillán, César R. García-Jacas, Yovani Marrero-Ponce

**Affiliations:** 1 International Max Planck Research School (IMPRS) for Molecular Biology, Georg-August Universität Göttingen, Justus-von-Liebig-Weg 11, 37077 Göttingen, Germany; k.castillomendieta@stud.uni-goettingen.de; 2 Interdisciplinary Centre of Marine and Environmental Research (CIIMAR), University of Porto, Terminal de Cruzeiros do Porto de Leixões, Av. General Norton de Matos, 4450-208 Porto, Portugal; 3 Department of Biology of Faculty of Sciences, University of Porto, Rua do Campo Alegre, 4169-007 Porto, Portugal; 4 Grupo de Investigaciones en Química y Biología, Facultad de Ciencias Exactas, Universidad del Norte, Carrera 51B, Km 5, vía Puerto Colombia, Barranquilla 081007, Colombia; ebrazon@uninorte.edu.co; 5 Grupo de Química Computacional y Teórica (QCT-USFQ), Departamento de Ingeniería Química, Universidad San Francisco de Quito, Diego de Robles y Vía Interoceánica, Quito 170901, Pichincha, Ecuador; jrmora@usfq.edu.ec; 6 Colegio de Ciencias e Ingenierías “El Politécnico”, Universidad San Francisco de Quito (USFQ), Quito 170157, Pichincha, Ecuador; nperez@usfq.edu.ec; 7 Programa de Química, Facultad de Ciencias Básicas, Universidad del Atlántico, Barranquilla 080003, Colombia; nestorcubillan@mail.uniatlantico.edu.co; 8 Investigador por México, Secretaría de Ciencia, Humanidades, Tecnología e Innovación (Secihti), Ciudad de México 03940, Mexico; cesarrjacas1985@gmail.com; 9 Unidad de Posgrado e Investigación, Instituto Tecnológico de Mérida, Tecnológico Nacional de México, Av. Tecnológico, Km. 4.5, Mérida 97000, Mexico; 10 Facultad de Ingeniería, Universidad Panamericana, Augusto Rodin No. 498, Insurgentes Mixcoac, Benito Juárez, Ciudad de México 03920, Mexico; 11 Universidad San Francisco de Quito (USFQ), Grupo de Medicina Molecular y Traslacional (MeM&T), Colegio de Ciencias de la Salud (COCSA), Escuela de Medicina, Edificio de Especialidades Médicas, and Instituto de Simulación Computacional (ISC-USFQ), Diego de Robles y vía Interoceánica, Quito 170157, Pichincha, Ecuador

**Keywords:** bioactive peptides, peptide chemical space, complex networks, molecular descriptors, chemical space networks, half-space proximal networks, multi-query similarity searching

## Abstract

Bioactive peptides constitute a highly diverse and therapeutically relevant molecular class, yet their systematic exploration remains challenging because of the vast size, heterogeneity, and fragmented annotation of peptide chemical space. In this context, complex networks have emerged as a complementary computational framework for organizing, analyzing, and exploiting peptide diversity. This methodological review examines the main components of graph-based peptide informatics, from graph-based data integration and curated repositories to descriptor-based representations, similarity-driven network construction, and topology-informed analysis. We describe how peptide sequences can be projected into multidimensional reference spaces using molecular descriptors, aggregation operators, and unsupervised feature selection, and how these representations support the construction of Chemical Space Networks, Half-Space Proximal Networks, and Metadata Networks. Special attention is given to topological analysis, including threshold selection, community detection, and centrality-based identification of representative peptides and scaffolds. We also review the development of Multi-query Similarity Searching Models as training-independent, topology-guided alternatives to conventional supervised predictors. Finally, we highlight the implementation of these methodologies in computational resources such as StarPepDB, StarPep Toolbox, and StarPepWeb, which illustrate the transition of peptide network science from conceptual workflows to accessible, scalable, and reproducible infrastructures. Overall, complex networks are presented as a mature and interpretable paradigm for the structured exploration, analysis, and discovery of bioactive peptides.

## 1. Introduction

Bioactive peptides constitute a highly diverse and therapeutically relevant class of molecules with broad biological activities, including antimicrobial, antiviral, antiparasitic, antitumor, antibiofilm and immunomodulatory functions [1,2]. Their growing relevance in drug discovery reflects their capacity to combine molecular specificity, structural versatility, and functional breadth across multiple therapeutic areas [1,2,3,4,5]. However, the systematic exploration of peptide chemical space remains a major conceptual and computational challenge [6,7,8,9]. Even when restricted to relatively short sequences, the theoretical peptide space is enormous, with estimates exceeding 10^65^ possible sequences for peptides of 50 residues or fewer [6,7,8,9]. Only a small fraction of this space has been experimentally explored, annotated, and organized into comprehensive databases, creating a substantial gap between theoretical diversity and curated knowledge available for computational discovery [10,11,12].

The rapid accumulation of bioactive peptide sequences in public repositories has created both opportunities and methodological difficulties. On the one hand, bioactive peptide sequences have been collected and organized into several specialized databases covering antimicrobial peptides–APD3, CAMPR3, DBAASP, DRAMP, and dbAMP [13,14,15,16,17]–as well as antiviral peptides (AVPdb [18]), antibiofilm peptides (BaAMPs [19]), hemolytic peptides (Hemolytik [20]), anticancer peptides (CancerPPD [21]) and tumor-homing peptides (TumorHoPe [22]), among many other peptide classes. Broader or integrative resources, including the Database of Food-derived Bioactive Peptides (DFBP), Peptipedia, and StarPepWeb, have also contributed to organizing peptide knowledge across different application domains [12,23,24].

As previously reported, the bioactive peptide database landscape remains heterogeneous because specialized resources are typically developed for different activities. Thus, even when data are compiled into integrative repositories, source-level differences in metadata structures, annotation vocabularies, curation criteria, and redundancy levels can still limit cross-database mining and large-scale comparative analyses [12,23,24]. Early comparative analyses of antimicrobial peptide databases revealed substantial overlap among repositories, supporting the need for non-redundant integration before robust large-scale analyses are undertaken [24]. This challenge is particularly relevant for multifunctional peptides, since the same sequence may appear across different resources under distinct biological labels, taxonomic origins, or experimental contexts.

**Figure 1:**
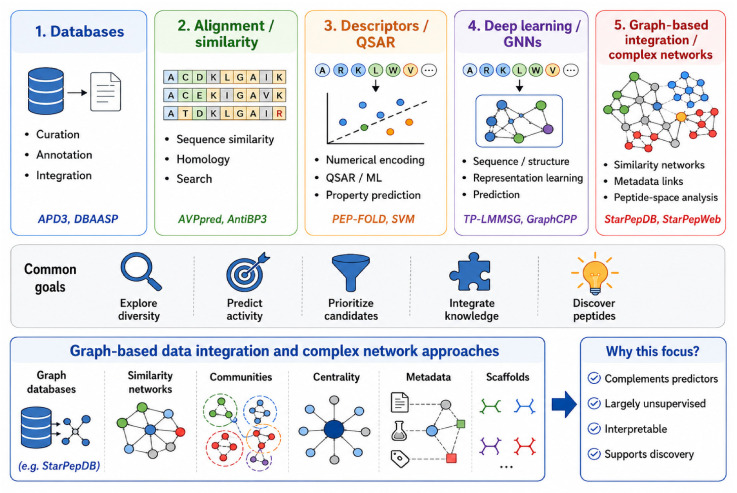
Computational strategies for bioactive peptide research and the scope of this review. Bioactive peptide data can be explored through databases, alignment/similarity-search methods, descriptor/QSAR models, deep-learning and GNN-based predictors, and graph-based complex network approaches. This review focuses on graph-based data integration and complex networks as interpretable frameworks for peptide-space organization, topological analysis, metadata-aware interpretation, scaffold selection, and similarity-driven discovery.

Computational strategies for bioactive peptide research can be broadly organized into several complementary families. Database-centered approaches focus on collecting, curating, and annotating peptide sequences, activities, structures, and metadata [10,11,12,13,14,15,16,17,18,19,20,21,22,23]. Alignment- and similarity-search-based methods exploit sequence similarity, local alignments, or homology relationships to identify peptides related to known bioactive sequences or to infer potential functions [25,26,27]. Descriptor-based and QSAR-like approaches transform peptides into numerical representations that can be used for statistical modeling and classical machine learning [28]. More recently, deep learning and graph-aware predictive models have enabled the use of sequence-, residue-, structure-, or multimodal representations for activity prediction and prioritization of bioactive peptides [29,30,31,32]. In parallel, graph-based data integration and complex network approaches organize peptides, similarities, and metadata as explicit relational systems, allowing the exploration of chemical space, connectivity, community structure, and representative bioactive scaffolds [6,23,33,34,35]. The present review focuses on this last family of approaches, while discussing its complementarity with other computational strategies (Figure 1).

Among the computational families summarized in Figure 1, supervised machine learning and deep learning have played a particularly prominent role in peptide activity prediction and have substantially contributed to peptide screening and prioritization [29,36]. However, their performance depends heavily on the quality, representativeness, and balance of the training data. A persistent limitation is the construction of reliable negative datasets, as experimentally validated inactive peptides are often scarce or unavailable. As a result, many predictors rely on random peptides, shuffled sequences, or loosely defined non-active sets, which may introduce selection bias, inflate performance estimates, and reduce biological interpretability [37,38]. These limitations do not diminish the value of supervised learning, but they highlight the need for complementary strategies capable of extracting structure, diversity, and functional relationships directly from peptide chemical space, including graph-based approaches that can organize known and unlabeled peptides without requiring predefined positive and negative classes.

Graph neural networks (GNNs), hybrid sequence–graph models, and related multimodal architectures represent another important development in this landscape. They have been increasingly applied to tasks such as activity classification, toxicity prediction, peptide–protein interaction modeling, and membrane permeability assessment [31,32,39]. These approaches are highly valuable for representation learning and predictive performance, especially when atom-level, residue-level, structural, or multimodal encodings are employed [40]. However, in most peptide applications, GNNs use graph representations as input objects for supervised modelling. This differs from the focus of the present review, which addresses explicit graph representations of peptide collections and metadata relationships, where the network itself is the object of analysis through similarity, topology, community structure, and node-level interpretation.

From this perspective, graph-based data integration and complex network science have emerged as complementary methodological frameworks for bioactive peptide research. Rather than treating peptides only as isolated sequences or labeled examples for prediction, graph-based approaches represent them as interconnected entities embedded within structured relational or similarity spaces [6,34]. This allows peptide collections to be analyzed through local proximity, global connectivity, community organization, metadata associations, and node influence. Consequently, complex network methodologies provide a largely unsupervised and interpretable route for exploring bioactive peptide diversity, identifying representative scaffolds, detecting atypical regions of chemical space, and supporting similarity-driven discovery and inference [35,41,42,43].

The development of graph-based peptide informatics has required not only methodological concepts, but also curated resources capable of supporting their practical application. StarPepDB represented an important implementation of graph-based peptide data integration by organizing heterogeneous peptide information from multiple public databases into a curated graph structure linking peptide sequences to metadata such as biological function, origin, source database, targets, modifications, and literature references [23]. More recently, StarPepWeb broadened access to StarPepDB through a browser-accessible platform that integrates 45,120 non-redundant peptide sequences from 40 public databases into a source-tracked graph enriched with metadata, physicochemical properties, predicted three-dimensional structures, and modern peptide representations [12]. In this review, StarPepDB, StarPep Toolbox, and StarPepWeb are discussed as representative implementations of the broader graph-based peptide informatics framework, rather than as the sole object of analysis.

Using curated and integrated peptide resources as starting points, descriptor-based peptide representations have enabled the construction of chemical reference spaces suitable for alignment-free similarity analysis [6,33]. In this framework, peptide sequences are transformed into numerical molecular descriptors derived from amino acid property vectors, aggregation operators, and feature-selection procedures. These descriptors allow peptides of different lengths and compositions to be compared as physicochemical objects within a common multidimensional space [6,33]. Chemical Space Networks (CSNs), Half-Space Proximal Networks (HSPNs), and Metadata Networks (METNs/MNs) then provide distinct graph abstractions of this space, each with specific practical advantages. CSNs emphasize dense similarity relationships and are useful for exploring local neighborhoods and community structure; HSPNs reduce network complexity while preserving relevant proximity relationships, facilitating scaffold extraction and large-scale visual analysis; and METNs/MNs connect peptide organization with biological context by linking sequences to metadata such as activities, origins, targets, or source databases. Together, these network archetypes support a multi-layered representation of peptide chemical space that combines structural similarity, topological organization, and metadata-aware interpretation [6,33,35,43].

Beyond visualization, complex network methodologies have enabled a transition toward topology-informed prediction. Community detection and centrality analysis are not only descriptive tools; they can also guide the extraction of representative and non-redundant peptide scaffolds [33,35,41,43]. These scaffolds can be used as query sets in Multi-query Similarity Searching Models (MQSSMs), which perform similarity-based screening through group fusion strategies such as the MAX-SIM rule [25,33,44]. Unlike conventional supervised classifiers, MQSSMs do not require model training in the classical sense and can reduce dependence on artificially constructed negative datasets. Applications to antiparasitic, tumor-homing, antiviral, and hemolytic peptides have shown that network-derived query sets can support robust and interpretable similarity-based prediction [25,33,42].

This review provides a methodological overview of graph-based architectures and complex network approaches applied to bioactive peptide research. Specifically, it examines the data integration principles underlying peptide graph databases, the descriptor-based definition of peptide chemical reference spaces, the main network archetypes used to model similarity and metadata relationships, the topological analyses used to interpret these networks, and the predictive frameworks derived from them. Representative implementations, including StarPepDB, StarPep Toolbox, and StarPepWeb, are discussed to illustrate how these concepts can be operationalized in practical computational environments. Overall, the review aims to position complex networks as an interpretable and complementary framework for organizing, exploring, and exploiting bioactive peptide chemical space [12,34].

## 2. The Foundation: Graph-Based Data Integration

### 2.1. Star Schema Architecture and Dimensional Modeling

A robust methodological framework for complex network analysis in bioactive peptide research requires the integration of heterogeneous peptide information into a structured and consistent data environment. This need arises from the fragmented nature of peptide knowledge, which is distributed across multiple specialized repositories, often with substantial overlap in sequence content, inconsistent annotation practices, and non-uniform terminologies [23]. Within this framework, StarPepDB plays a central role as a curated and integrated graph database that unifies 45,120 unique peptide sequences collected from 40 diverse sources [23].

**Figure 2:**
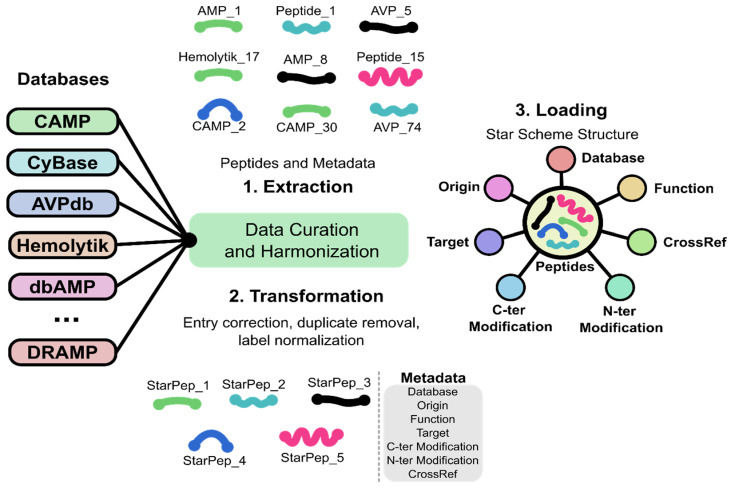
ETL workflow for the integration of peptide information into a standardized graph representation. Peptide sequences and associated metadata were extracted from 40 source databases, transformed through curation and harmonization steps, and finally loaded into a graph database following a star schema organization. During transformation, malformed entries were corrected, duplicate peptides were removed, and metadata labels were standardized.

The internal organization of StarPepDB follows a star schema architecture [45], in which peptides are the core entities connected to metadata information in a star- or asterisk-like shape (Figure 2). This additional information describes the peptide’s source databases, function, source organisms, biological targets, chemical modifications and cross-references to entries in UniProtKB [46], PDB [47] and PubMed [48]. This design is methodologically relevant because it separates the central sequence object from its associated annotation layers while preserving their relationships in a coherent graph structure [23].

### 2.2. Data Curation and Harmonization

The integration of peptide information into StarPepDB depends on a structured Extract, Transform, Load (ETL) workflow. This process is responsible for collecting data from multiple repositories, transforming them into a common internal representation, and loading them into the final graph architecture after curation and harmonization (Figure 2). This step is essential because the quality of any network model derived from the database is directly conditioned by the quality of the underlying integrated data.

The extraction phase involves gathering peptide records and associated metadata from distinct sources. The transformation phase is the most critical from a methodological standpoint, as it includes the correction of malformed entries, the identification and removal of duplicates, the normalization of descriptors and labels, and the reconciliation of heterogeneous nomenclatures [23]. For instance, StarPepDB addresses this nomenclature variability by incorporating semantic standardization through hierarchical relationships, particularly the use of the “is_a” relationship to organize taxonomic and functional information. In taxonomic metadata, this strategy enables the representation of hierarchical ranks following the Integrated Taxonomic Information System (ITIS).

In functional metadata, it allows more specific activity annotations to be nested under broader categories; for instance, terms such as anti-Gram-positive can be represented as subordinate to the broader antibacterial category [23]. Finally, the loading phase inserts the curated information into the graph database according to the star schema structure. Through this workflow, raw repository content is converted into a coherent analytical resource, suitable for chemical space modeling and graph-based exploration.

### 2.3. Methodological Significance of Graph-Based Integration

The importance of graph-based data integration extends beyond database design. By organizing sequences and metadata within a unified, relational structure, this approach creates the conditions required for the rest of the analytical pipeline. Descriptor-based representations, similarity calculations, metadata networks, and topology-driven predictive models all depend on the availability of a curated and semantically coherent reference space. In other words, reliable complex network analysis of bioactive peptides is only possible if the input data have already been normalized at both the sequence and annotation levels.

From a methodological perspective, StarPepDB not only acts as a repository that centralizes peptide knowledge from multiple sources, but it also provides the relational infrastructure needed to transform fragmented peptide information into a navigable chemical space. For these reasons, graph-based data integration should be regarded as the foundational layer of the entire methodological ecosystem. It is at this stage that raw peptide data are converted into a structured reference space from which complex network representations can be built in a consistent and analytically meaningful manner.

## 3. Defining the Chemical Reference Space

Once peptide information has been curated and integrated into a coherent graph-based repository, the next methodological step is to define a numerical representation of peptide space suitable for similarity analysis.

Peptide information can be encoded in different ways depending on the analytical objective, including primary sequences, amino acid or dipeptide composition, k-mer frequencies, one-hot encodings, learned embeddings, structural descriptors, activity labels, and metadata-based representations. External tools and platforms such as iFeature, iFeatureOmega, Pfeature, and ProtSpace further illustrate the diversity of available feature-encoding, embedding, and visualization strategies for protein and peptide sequence spaces [49,50,51,52]. In the workflow reviewed here, the focus is specifically on descriptor-based encoding, where peptides are transformed into fixed-length numerical vectors derived from sequence-related physicochemical properties.

**Figure 3:**
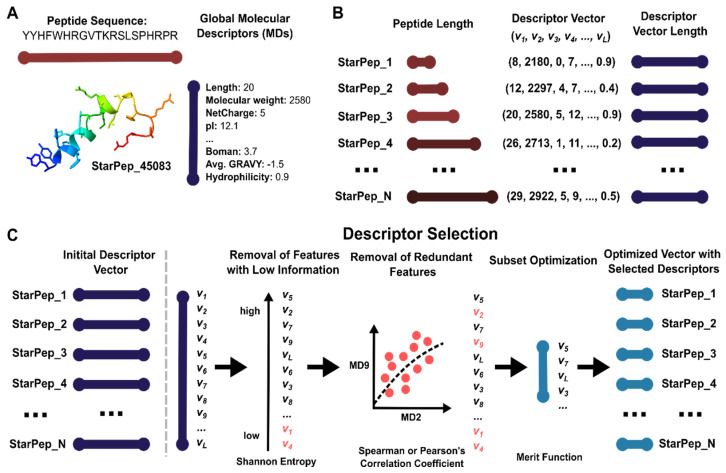
Descriptor-based representation of peptides and unsupervised refinement of the chemical reference space. (**A**) Peptides can be encoded through global molecular descriptors that summarize sequence-derived physicochemical properties, such as molecular weight, net charge, isoelectric point (pI), hydrophobicity-related indices, and aggregation-derived descriptors. The peptide StarPep_45083 is shown as an illustrative example. Its 3D structure was generated with AlphaFold 3 for visual reference only [53]. (**B**) Because peptide sequences vary in length, descriptor-based encoding transforms each peptide into a fixed-length numerical vector suitable for comparison across a large dataset. (**C**) The initial descriptor space is refined through a two-stage unsupervised workflow involving removal of low-information descriptors, redundancy control by correlation filtering, and subset optimization based on entropy- and mutual-information-related criteria. Low-information descriptors are numerical features with limited discriminatory value across the peptide dataset. This process yields optimized descriptor vectors with selected, informative, and minimally redundant features for similarity estimation and network construction.

Because peptide sequences vary in length, composition, and physicochemical organization, they cannot be directly compared in a unified analytical framework without first being transformed into comparable mathematical objects [6,7]. For this reason, bioactive peptides are projected into a multidimensional chemical reference space in which each sequence is represented by a vector of molecular descriptors (Figure 3A,B) [6,7,28]. This transformation is essential for subsequent alignment-free similarity estimation and for the construction of graph models that capture the structure of peptide chemical space [6,7].

As summarized in the original workflow, this stage relies on: (i) descriptor calculation from amino acid property vectors (v), (ii) unsupervised feature selection based on entropy and redundancy control, and (iii) generation of similarity relationships from the resulting descriptor space [6,34].

### 3.1. From Peptide Sequences to Descriptor-Based Embeddings

The definition of a chemical reference space begins with the conversion of symbolic peptide sequences into quantitative embeddings [6,7]. Let the i-th peptide sequence be denoted by

$$S_{i}={\left(a\right.}_{1},\;a_{2},\ldots ,a_{L_{i}})$$

where $L_{i}\;$ is the sequence length and each $a_{r}$ is an amino acid residue. If each residue is associated with a q-dimensional physicochemical property vector ${Ρ(a}_{r})\in \;R^{q}$, then a descriptor mapping ϕ  transforms the variable-length sequence $S_{i}$ into a fixed-length vector

$$x_{i}=\phi \left(S_{i}\right)\in R^{m}.$$

The complete peptide collection is therefore represented by a descriptor matrix

$$D={\left[x_{ij}\right]}_{n\times m},\;$$

where n is the number of peptides and m is the number of calculated molecular descriptors [6]. This formalization is consistent with the descriptor-space framework used in the original similarity-network workflow, where peptide instances occupy the rows of the matrix and descriptor variables occupy its columns [6].

Unlike local alignment approaches, which focus on residue-to-residue correspondence, descriptor-based embeddings provide a higher-level representation of the entire molecule [6]. This makes them particularly suitable for large-scale exploration of peptide collections, where the aim is not only to assess sequence homology, but also to identify broader patterns of physicochemical and functional similarity [7,28]. Within the methodological framework reviewed here, these embeddings constitute the substrate for the later construction of similarity-based networks and the identification of representative regions of peptide chemical space [6,7].

### 3.2. Molecular Descriptors for Global Peptide Characterization

To generate these embeddings, peptides are characterized through molecular descriptors derived from amino acid property vectors [6,54]. These descriptors aim to capture relevant aspects of peptide composition and physicochemical behavior in a compact and computable form. Rather than describing residues in isolation, the framework emphasizes global peptide characterization, in which residue-level information is integrated into sequence-level summaries (Figure 3A) [6].

This strategy is especially appropriate for bioactive peptides because many relevant activities depend on emergent molecular patterns—such as overall charge distribution, hydrophobic balance, amphipathic tendency, or conformational propensity—rather than on simple sequence identity alone [28,55]. Descriptor-based characterization therefore enables peptides to be compared as physicochemical objects embedded in a common reference space, supporting both exploratory analysis and downstream similarity-based inference [6,7].

In practice, the descriptor families used in this framework extend beyond conventional amino acid composition and include descriptors derived from amino acid properties, residue classes, and aggregation operators. The original implementation explicitly states that hundreds or even thousands of such descriptors can be generated from peptide sequences by combining amino acid property sets with statistical and aggregation-based summarization schemes [6,34].

### 3.3. Aggregation Operators and Descriptor Compression

A key methodological component of this representation step is the use of statistical and aggregation operators to transform residue-level property profiles into fixed-length descriptor vectors [6,34]. If a physicochemical property profile along a peptide is represented by

$$v={\left(v\right.}_{1},\;v_{2},\ldots ,v_{L}),$$

then classical global summaries include the arithmetic mean.

$\;\mu \left(v\right)=\frac{1}{L}\sum_{r=1}^{L}v_{r},\;$ and the standard deviation

$$\sigma \left(v\right)=\sqrt{\frac{1}{L}\;\sum_{r=1}^{L}{\left(v_{r}-\mu \left(v\right)\right)}^{2}}.\;$$

More flexible operators can also be used to capture different aspects of the internal sequence organization. A standard example is the ordered weighted averaging (OWA) operator,

$$OWA\left(v\right)=\sum_{r=1}^{L}w_{r}v_{\left(r\right)},$$

where $v_{\left(r\right)}$ denotes the reordered values of the profile and the weights satisfy $w_{r}\geq 0$ and $\sum_{r=1}^{L}w_{r}\;=1$. By assigning different weights to ordered positions, OWA allows the descriptor to emphasize high, intermediate, or low local values rather than simple central tendency [56].

More general schemes, such as the discrete Choquet integral, allow interactions among residue-level contributions to be incorporated beyond simple linear averaging [57,58]. In the StarPep framework, sequence-based molecular descriptors are generated by applying statistical, fuzzy, and non-fuzzy aggregation operators to amino acid physicochemical properties, thereby projecting peptides into a multidimensional descriptor space suitable for similarity analysis [6,34].

This compression step is essential because peptide sequences are inherently variable in length, and a direct comparison of raw residue-wise profiles is not always feasible in large-scale analyses (Figure 3B). By reducing variable-length sequences to fixed-dimensional vectors, these operators enable computationally tractable similarity estimation across large peptide collections while preserving relevant aspects of physicochemical organization [6,34].

### 3.4. Unsupervised Feature Ranking by Shannon Entropy

Because descriptor generation may produce hundreds of variables, an additional methodological step is required to identify the most informative ones (Figure 3C) [59]. In the framework reviewed here, the first stage of unsupervised feature selection is based on Shannon entropy, which quantifies the variability and information content of each descriptor [6,60].

For a discretized descriptor $f_{j}$ with empirical bin probabilities $p_{jb}$, Shannon entropy is defined as

$$H\left(f_{j}\right)=-\sum_{b=1}^{B}p_{jb}{log}_{2}p_{jb}.$$

Descriptors with higher entropy are interpreted as having greater ability to discriminate among structurally different peptides, whereas low-entropy descriptors are less informative. In the original workflow, descriptors were ranked according to entropy, and low-information descriptors were removed using an entropy cutoff proportional to the maximum entropy of the dataset [6,60].

This criterion is particularly appropriate in an unsupervised setting because it does not depend on class labels or predefined prediction tasks. Instead, it captures the intrinsic diversity structure of the peptide collection itself, thereby providing a first filter for descriptors likely to support informative similarity relationships [6,7].

### 3.5. Redundancy-Aware Optimization Using Mutual Information and Correlation Filtering

Ranking descriptors by entropy alone is not sufficient, since highly informative variables may still carry overlapping information. For this reason, descriptor reduction in the StarPep workflow includes an explicit redundancy-control stage [6,34]. At a preliminary level, redundancy can be filtered by pairwise correlation (Figure 3C), using either Pearson’s or Spearman’s coefficient to identify strongly associated descriptors [61,62]. In the methodological assessment reported for the original workflow, both criteria were explored at different thresholds, and a Spearman-based filter was ultimately selected as the most appropriate option for generating the candidate descriptor set. More specifically, the preliminary analyses examined high-correlation cutoffs such as 0.95, whereas the subsequent ranking-and-filtering study identified Spearman correlation with a threshold of 0.8 as a suitable compromise between redundancy reduction and preservation of the original data structure [6].

In information-theoretic terms, dependency between two descriptors $f_{j}$ and $\;f_{k}$ can be quantified by mutual information [60]:

$$I\left(f_{j};f_{k}\right)=\sum_{u}\sum_{\upsilon }p_{jk}\left(u,\;\upsilon \right){log}_{2}\frac{p_{jk}\left(u,\;\upsilon \right)}{p_{j}\left(u\right)\;p_{k}\left(\upsilon \right)}.$$

Large values of *I* $\left(f_{j};\;f_{k}\right)$ indicate that the two features share information and may therefore be partially redundant. The second stage of the StarPep feature-selection pipeline further refines the candidate set by applying subset optimization based on entropy and mutual information concepts [6,34,59].

Conceptually, the goal is to maximize information content while minimizing redundancy, which can be expressed through a merit function of the form

$$J\left(S\right)=\frac{1}{\left|S\right|}\sum_{f_{j}\;\in \;S}H\left(f_{j}\right)-\lambda \frac{1}{\left|S\right|\left(\left|S\right|-1\right)}\sum_{\begin{array}{c} f_{j},\;f_{k\;}\in \;S\\ j\neq k\; \end{array}}I\left(f_{j},f_{k}\right),$$

where λ controls the trade-off between relevance and redundancy [59]. In practice, the original study reported a feature subset optimization step that reduced the descriptor space to compact subsets, often in the range of 30–50 descriptors, suitable for subsequent network construction [6].

Thus, descriptor selection in this framework should be understood as a two-layer process: first, the removal of low-information and highly correlated variables; and second, the optimization of a compact subset that preserves discriminatory capacity while avoiding unnecessary redundancy [6,34,59].

### 3.6. Descriptor Selection as a Prerequisite for Alignment-Free Similarity Networks

The outcome of this stage is a curated multidimensional reference space in which peptides are represented through selected descriptors with high information content and controlled redundancy [6,34]. This reference space is not an end, but a prerequisite for the next analytical step: the construction of alignment-free similarity networks [6,7].

Once peptide embeddings $x_{i}$ and $x_{k}$ are defined, pairwise relationships can be estimated through a distance or similarity function,

$$d_{ik}=d\left(x_{i},\;x_{k}\right),s_{ik}=s\;\left(x_{i},\;x_{k}\right).$$

The optimized sets of molecular descriptors implemented in StarPep Toolbox v0.8.5 are explicitly intended to enable the computation of similarity indices, clustering analyses, and correlation or similarity networks from peptide sequence data [34].

This is a critical conceptual point in the methodological pipeline. The reliability of chemical space networks, half-space proximal networks, and related topological analyses depends directly on how well the descriptor space captures meaningful physicochemical relationships among peptides [6,33,43]. If the descriptor representation is noisy, redundant, or poorly informative, the induced topology will also be distorted. Conversely, a well-defined chemical reference space provides the mathematical substrate needed for robust similarity estimation and meaningful graph-based modeling [6,7].

For this reason, the definition of the chemical reference space should be regarded as a central methodological bridge between graph-based data integration and complex network construction. It is at this stage that curated peptide knowledge is transformed into a quantitative representation capable of supporting similarity-driven exploration of bioactive peptide landscapes [6,7,34].

## 4. Complex Network Archetypes

Once peptides have been embedded into a curated multidimensional reference space, the next methodological step is to represent their relationships through graph models capable of capturing the organization of peptide chemical space [6,7]. In general terms, a network can be represented as a graph.

$$G=\left(V,\;E\right),$$

where V is the set of vertices, also referred to as nodes in network terminology, and E is the set of edges connecting them [63]. In graph-based peptide informatics, nodes typically represent peptides or metadata entities, whereas edges encode relationships derived from descriptor-based similarity, geometric proximity, or biological annotation [6,23]. In this context, complex networks function as abstractions of peptide space that enable the transition from pairwise similarity calculations to interpretable topological organization [6,64].

The construction of these networks depends directly on the choice of the underlying similarity or distance function used to quantify pairwise relationships between peptide embeddings [6,7,34]. In practice, different alignment-free measures may emphasize distinct aspects of descriptor-space organization, and therefore influence the number of retained edges, the preservation of local neighborhoods, and the overall topology of the resulting graph. In the StarPep Toolbox framework, network construction can rely on several metrics, including Euclidean, Manhattan, Chebyshev, Canberra, Soergel, Bhattacharyya, and Angular separation, among others [34]. For this reason, (dis)similarity metrics should be regarded as a methodological layer connecting descriptor-based peptide representations with the network archetypes built upon them, rather than as a purely technical parameter choice [6,7,34].

**Figure 4:**
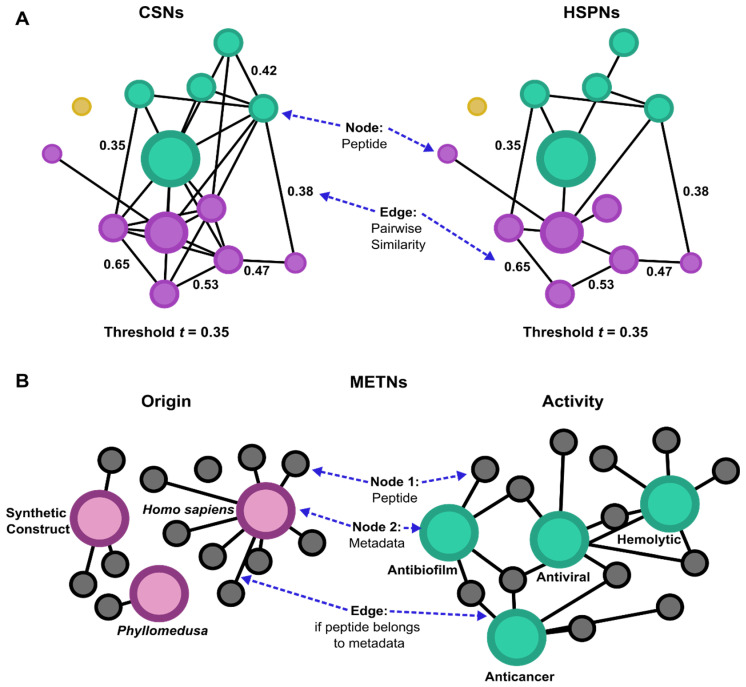
Main complex network archetypes used in graph-based peptide informatics. (**A**) Similarity networks represented by Chemical Space Networks (CSNs) and Half-Space Proximal Networks (HSPNs). CSNs are dense, threshold-based similarity graphs in which nodes represent peptides and edges retain pairwise similarities above a predefined cutoff. HSPNs are sparse proximity graphs that preserve only a reduced subset of informative neighborhood relations, thereby minimizing edge redundancy while retaining the geometric backbone of peptide space (**B**) Metadata Networks (METNs/MNs) are bipartite or pseudo-bipartite graphs that connect peptide nodes to metadata categories such as origin, activity, target, or source database. Unlike CSNs and HSPNs, edges in METNs/MNs encode annotation-based associations rather than chemical similarity. Together, these archetypes provide complementary views for mapping peptide similarity landscapes, extracting simplified proximity backbones and representative scaffolds, and interpreting peptide clusters through biological metadata.

Different network archetypes have been introduced to address distinct analytical needs, ranging from dense representations of global similarity patterns to sparse proximity-preserving structures and metadata-driven contextual maps. Rather than being mutually exclusive, these models should be understood as complementary views of bioactive peptide space, each emphasizing different practical uses in peptide analysis and discovery (Figure 4) [33,35,41,43].

### 4.1. Chemical Space Networks (CSNs)

Chemical Space Networks (CSNs) constitute one of the most established and intuitive graph representations for analyzing chemical space [65]. Formally, a CSN can be written as a graph $G=\left(V,\;E\right)$, where each node $\nu_{i}\;\in V$ represents a peptide and an edge $e_{ij}\;\in E$ is established between two peptides whenever their pairwise similarity $s_{ij}$ exceeds a predefined threshold t,

$$e_{ij}\;\in \;E\;⟺\;s_{ij}\;\geq \;t.$$

This relationship can be expressed in matrix form through a threshold-based adjacency matrix,

$$A_{ij}=\left\{\begin{array}{c} 1,\;s_{ij}\geq t\\ 0,\;s_{ij}<t \end{array}\right.$$

or, in the weighted version,

$$W_{ij}=\left\{\begin{array}{c} s_{ij},\;s_{ij}\geq t\\ 0,\;s_{ij}<t \end{array}\right.$$

In the context of bioactive peptide analysis, the similarity values are typically derived from alignment-free comparisons performed in descriptor space, allowing the chemical landscape to be explored independently of direct residue-to-residue alignment [6,34]. The resulting network is usually dense and weighted, with edge weights reflecting the strength of pairwise similarity relationships [6,65].

The principal value of CSNs lies in their ability to map peptide-space organization at a broad scale. In practical terms, this allows researchers to identify densely connected similarity neighborhoods, communities of related peptides, highly connected regions, bridge-like peptides, and isolated sequences. CSNs are therefore useful for exploratory graph mining, community detection, similarity-based functional hypotheses, and the selection of representative peptides according to their local and global positions within the network [6,33].

At the same time, dense connectivity can become a methodological limitation as the dataset size increases. Very large numbers of edges may hinder visualization, increase computational cost, and reduce interpretability if the similarity threshold is not carefully selected [6,65]. Thus, CSNs are best understood as informative but potentially redundant representations of peptide space: they maximize relational richness, but this same property can complicate downstream topological analysis when the graph becomes excessively dense [6,33].

### 4.2. Half-Space Proximal Networks (HSPNs)

To address some of the limitations associated with dense threshold-based graphs, Half-Space Proximal Networks (HSPNs) have been introduced as a sparse alternative for modeling peptide chemical space [35,44,66]. Unlike Chemical Space Networks, which retain all pairwise relationships above a predefined similarity threshold, HSPNs aim to preserve only a reduced subset of informative neighborhood connections while maintaining the essential geometric organization of the descriptor space. In methodological terms, HSPNs belong to the broader family of proximity graphs and are constructed through the Half-Space Proximal (HSP) test originally proposed for sparse graph extraction [66].

Let $V=\left\{v_{1},\;\ldots ,\;v_{n}\right\}$ denote the peptide set embedded in descriptor space. For a given peptide $v_{i}$, the HSP procedure iteratively selects proximal neighbors and excludes candidate nodes falling into the forbidden half-space induced by previously selected neighbors. The resulting edge set defines a sparse graph $G_{HSPN}=\;\left(V,\;E_{HSPN}\right)$ where $E_{HSPN}$ represents a reduced subset of proximity-preserving edges over the complete node set.

In contrast to CSNs, whose density is mainly controlled by a global similarity cutoff, HSPNs derive sparsity from a geometric neighborhood rule and are therefore not simple threshold-pruned versions of dense similarity graphs [66]. This distinction is illustrated schematically in Figure 4A, where CSNs retain a denser set of above-threshold similarity relations, whereas HSPNs preserve a reduced subset of informative neighborhood connections.

Within graph-based peptide informatics, HSPNs have proven useful as compact representations of peptide chemical space. Rather than retaining all pairwise similarities, they preserve a simplified backbone of the most informative neighborhood relationships, making them especially suitable for large peptide collections, reduced-noise visualization, representative scaffold extraction, similarity-driven candidate prioritization, and predictive frameworks such as MQSSMs [25,41,44].

### 4.3. Metadata Networks (METNs/MNs)

Whereas CSNs and HSPNs focus on similarity relationships among peptides, Metadata Networks (METNs), also termed as MNs, extend the analysis by incorporating the biological and informational context associated with peptide sequences [23,35]. Formally, these networks are typically represented as bipartite or pseudo-bipartite graphs,

$$G=(V_{p}\;\cup \;V_{m},E),\;V_{p}\cap \;V_{m}=\emptyset ,$$

where $V_{p}$ denotes peptide nodes, $V_{m}$ denotes metadata nodes, and edges connect peptides to categories such as taxonomic origin, biological activity, target organism, or source database. Thus, edges in METNs do not encode chemical similarity, but annotation-based associations between peptides and their contextual descriptors [23,63]. A schematic representation of this peptide–metadata structure is shown in Figure 4B.

The methodological value of METNs/MNs lies in their interpretive power. While similarity networks reveal how peptides relate to one another in descriptor space, METNs/MNs help explain what those groupings may mean biologically by connecting peptides with metadata such as taxonomic origin, source organism, biological activity, target, or source database. For example, communities identified in a CSN or HSPN can be further examined through METNs/MNs to determine whether they are enriched in particular taxa, functional classes, biological sources, or database annotations. In this way, METNs/MNs provide a contextual layer that links chemical organization with biological interpretation, supporting the analysis of annotation patterns, functional overlap, metadata enrichment, and, when suitable taxonomic or source-organism information is available, evolutionary or ecological relationships within chemically coherent peptide clusters [33,35,41].

### 4.4. Dense Versus Sparse Representations of Peptide Chemical Space

A central methodological distinction within this framework concerns the trade-off between relational richness and structural parsimony. Although CSNs and HSPNs are both similarity-driven network models, they retain markedly different amounts of information. CSNs preserve a broad set of above-threshold similarity relationships and therefore provide a richer view of global connectivity, which is advantageous for exploratory analyses and for detecting large-scale organizational patterns in peptide space. However, this same richness may generate visually crowded and computationally expensive graphs as the dataset size increases [6,65].

By contrast, HSPNs preserve only a reduced set of informative neighborhood relations, thereby emphasizing the geometric backbone of peptide space rather than its full relational density. This makes them more suitable for large-scale visualization, reduced-noise topological analysis, and scaffold-centered applications in which redundant edges add limited analytical value. Accordingly, the choice between dense and sparse representations should be guided by analytical purpose rather than by methodological superiority: CSNs maximize relational completeness, whereas HSPNs emphasize tractability, clarity, and interpretability [6,33,43,65,66].

### 4.5. Complementary Roles of CSNs, HSPNs, and METNs

Taken together, CSNs, HSPNs, and METNs define a multi-layered graph-based framework for bioactive peptide analysis. Their complementarity lies in the fact that no single representation captures all relevant aspects of peptide space: similarity networks organize chemical relationships, whereas metadata networks provide the contextual layer needed for biological interpretation. By combining these representations, peptides can be analyzed simultaneously as chemical entities, topological objects, and annotated biological molecules.

For this reason, the use of multiple network archetypes should be regarded as one of the main strengths of graph-based peptide informatics. CSNs are better suited for broad exploration of similarity organization; HSPNs are better suited for simplified proximity analysis, scaffold selection, and candidate prioritization; and METNs/MNs are better suited for connecting peptide organization with biological annotations such as activity, origin, target, source database, or taxonomic information. Together, these complementary views provide the structural basis for downstream analyses such as community detection, centrality-based ranking, metadata-aware interpretation, and representative scaffold extraction [6,33,35,43].

## 5. Network Analytics and Topology

The construction of peptide similarity and metadata networks provides a structural representation of bioactive peptide space, but their full methodological value emerges only after topological analysis is applied to the networks. This allows researchers to evaluate how peptide space is structured, how communities of related peptides are formed, and which nodes occupy influential positions within the overall network architecture. Therefore, topological parameters are not merely descriptive statistics; they are operational tools for identifying informative similarity thresholds, detecting chemically coherent modules, and selecting representative peptides for downstream analysis.

### 5.1. Similarity Threshold Selection

A crucial step in the analysis of similarity-based peptide networks is the selection of an appropriate similarity threshold. If the threshold is too low, the resulting network may become excessively dense, obscuring meaningful modular structure and reducing interpretability. Conversely, if the threshold is too high, the graph may fragment into disconnected components, leading to the loss of relevant relationships among peptides [6,67].

**Figure 5:**
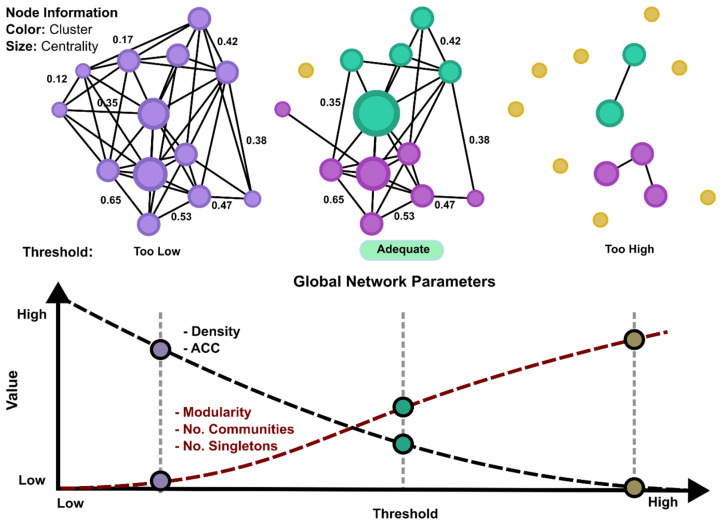
Selection process to get informative networks based on an adequate selection of an edge threshold value. The emergent topology of the graph is then assessed considering network parameters such as density, average clustering coefficient, modularity, number of communities and singletons. A very low threshold may lead to an excessively dense network, hindering meaningful modular structure and reducing interpretability. Conversely, if the threshold is too high, the graph may fragment into disconnected components, leading to the loss of relevant relationships among peptides. Once an adequate threshold has been identified, the topology of the network can be further studied by identifying and characterizing peptide clusters/communities. Furthermore, the relevance of each peptide within the network can be highlighted using different centrality metrics. In this scheme, the centrality of each peptide is represented by its node size, and clusters are represented by different node colors.

The optimal cutoff can be explored by monitoring global network properties such as density, modularity, the average clustering coefficient (ACC), diameter, and the number of communities and singletons (Figure 5).

Density is defined as the proportion of edges present in the network with respect to the total number of possible edges in the graph G=(V, E)

$$density\;\left(G\right)=\frac{2m}{n\left(n-1\right)},$$

where m=|E| and n=|V|. The density of a similarity network is inversely proportional to its threshold. An adequate density range has been proposed to fall between 0.01 and 0.25 in similarity networks [6,67].

CSNs are threshold-dependent graphs because the number of retained edges is directly determined by the cutoff applied. A CSN with threshold t=0 has density = 1, because all peptides are considered similar and are connected to each other, becoming the so-called complete graph [6,68]. Complete graphs do not provide relevant information about the topology of the peptide space; therefore, the selection of an adequate threshold is necessary for informative CSNs. Conversely, HSPNs are low-density networks even without the need to set a global similarity threshold.

Modularity quantifies the extent to which a network is partitioned into well-defined communities. A network with high modularity is dense within communities but sparse between them [69,70]. In weighted networks, modularity is commonly defined as follows:

$$Q=\frac{1}{2w}\;\sum_{i,j}\left(A_{ij}-\frac{k_{i}k_{j}}{2w}\right)\delta \left(c_{i}c_{j}\right),$$

where $A_{ij}$ is the weighted adjacency matrix entry between nodes i and j, $k_{i}=\sum_{j}A_{ij}$ is the strength of node i, $c_{i}$ is the community to which node i is assigned, and 2$w=\;\sum_{ij}A_{ij}$ is the total weight of the network. The term $\delta (c_{i},c_{j})$ takes the value of 1 if nodes i and j belong to the same community, and 0 otherwise [6,71]. Remarkably, HSPNs show higher modularity than CSNs even at low thresholds [33,41,43,44].

Communities or clusters or are subsets of nodes that are more strongly connected among themselves than to the rest of the graph, whereas singletons are isolated nodes that are not connected to any other node in the network. Communities from reported CSNs and HSPNs have commonly been identified using the Louvain method [69]. This method relies on the maximization of modularity using an iterative two-step process. First, it considers each node as its own cluster and calculates the increase in modularity by moving nodes to neighboring communities. Subsequently, these communities become super-nodes and the first step is repeated again until modularity no longer increases [69]. Community detection is especially relevant in peptide research because it enables the identification of families of peptides sharing similar physicochemical profiles or related topological positions. Rather than relying on predefined labels, this strategy allows clusters to emerge directly from the structure of the network itself.

The ACC quantifies the tendency of a network to form locally interconnected neighborhoods. The local clustering coefficient compares the number of edges among the neighbors of a given node with the total number of possible edges among those neighbors, and is defined as follows:

$$C_{i}=\frac{2e_{i}}{d(i)\left(d\left(i\right)-1\right)}$$

where $e_{i}$ is the number of edges among neighbors of node i, and d(i) is the degree of node i. The ACC of a network is obtained by averaging the local clustering coefficient over all nodes. The maximum peak of the ACC has been proposed as a useful indicator for identifying an appropriate threshold value t, as it often correlates with improved community structure in similarity networks [67].

The diameter represents the longest shortest-path distance between any two nodes in the graph. Formally, the diameter of a graph G=(V, E) is defined as follows:

$$diam\left(G\right)=\max_{i,j\in V}d(i,j),$$

where d(i,j) is the shortest-path distance between nodes i and j [68]. In practice, when the graph is disconnected, this measure is typically evaluated on the largest connected component. Networks with a small diameter represent compact graphs in which nodes can be reached in relatively few steps.

Together, these measures help identify thresholds that balance connectivity and structure, thereby producing networks with interpretable community organization and reduced topological noise. From a methodological standpoint, threshold analysis is fundamental because all subsequent topological inferences depend on the graph produced at this stage. A poorly chosen threshold may distort community boundaries, centrality rankings, and scaffold selection [67].

### 5.2. Centrality Metrics for Representative Peptide Identification

Centrality analysis focuses on the relative importance of individual nodes within the network. Influential peptides are ranked using several complementary centrality measures, including Weighted Degree (WD), Harmonic Centrality (HC), and Community Hub-Bridge (HB). Each of these metrics captures a different aspect of node relevance and therefore contributes a distinct perspective to the identification of representative peptides.

Weighted Degree (WD) reflects the strength of a node’s local connectivity by considering the number and weights of its adjacent edges.

In peptide similarity networks, nodes with high WD are typically embedded in densely connected local neighborhoods and may represent central members of chemically cohesive regions. WD centrality is defined as follows:

$${WD}_{i}=\sum_{j=1}A_{ij},$$

where $A_{ij}$ is the weight of the edge between nodes i and j. If no connection exists between two nodes, $A_{ij}=0$ [72].

Harmonic Centrality (HC), in contrast, captures a more global notion of prominence by considering how close a node is to all other nodes in the network. Peptides with high HC can therefore be interpreted as globally accessible or topologically influential nodes within the broader graph structure. HC is defined as follows:

$${HC}_{i}=\sum_{j\neq i}\frac{1}{d_{G}(i,j)},$$

where the geodesic distance $d_{G}(i,j)$ is the length of the shortest path from node i to node j [73].

Finally, Community Hub-Bridge Centrality (HB) emphasizes nodes that combine strong importance within their own community with a bridging role across different modules. These peptides are particularly interesting because they may function both as intra-community nuclei and as connectors between chemically distinct subspaces. HB centrality is defined as follows:

$${HB}_{i}=k_{i}^{int}*card\left(c_{i}\right)+k_{i}^{ext}*nnc\left(i\right),\;$$

where $k_{i}^{int}$ is the internal strength of node i, defined as the sum of the weights of the edges connecting node i to nodes within its own community; $k_{i}^{ext}$ is the external strength of node i, defined as the sum of the weights of the edges connecting node i  to nodes outside its community; $card\left(c_{i}\right)$ is the size of the community to which the node i belongs to; and $nnc\left(i\right)$ is the number of distinct communities that node i can reach through its inter-community links [74].

The use of multiple centrality measures is methodologically advantageous because peptide relevance is not a single-dimensional concept. A peptide may be highly central within a local module, broadly influential across the network, or strategically positioned between communities. By considering several centrality criteria jointly, the framework achieves a more balanced and informative selection of representative nodes for downstream exploration.

### 5.3. From Topological Descriptors to Chemical and Functional Interpretation

A major strength of this analytical framework is that topological descriptors can be translated into chemically and functionally meaningful interpretations. Density, clustering, modularity, and centrality are not treated simply as abstract graph measures; rather, they are used to infer how peptide space is organized, how similarity relationships concentrate within specific regions, and which peptides may serve as representative scaffolds or transitional entities [33,35,41,43].

For example, a network with strong modularity and well-defined communities suggests that the chemical space is partitioned into structured subdomains rather than being randomly organized. High clustering may indicate compact neighborhoods of closely related peptides, whereas nodes with high HB centrality may point to peptides located at the interface of different functional or structural groups. In this way, topological analysis provides a layer of interpretation that complements descriptor-based similarity and metadata annotation [6,33].

This interpretive step is important because it transforms the network from a visual object into an analytical model of peptide organization. It is through this translation from topology to meaning that complex networks become useful not only for exploration, but also for guiding similarity searches, scaffold extraction, and hypothesis generation [33,35,41,43,44].

## 6. Predictive Frameworks: Multi-Query Similarity Searching Models (MQSSMs)

### 6.1. Community-Informed Scaffold Extraction

**Figure 6:**
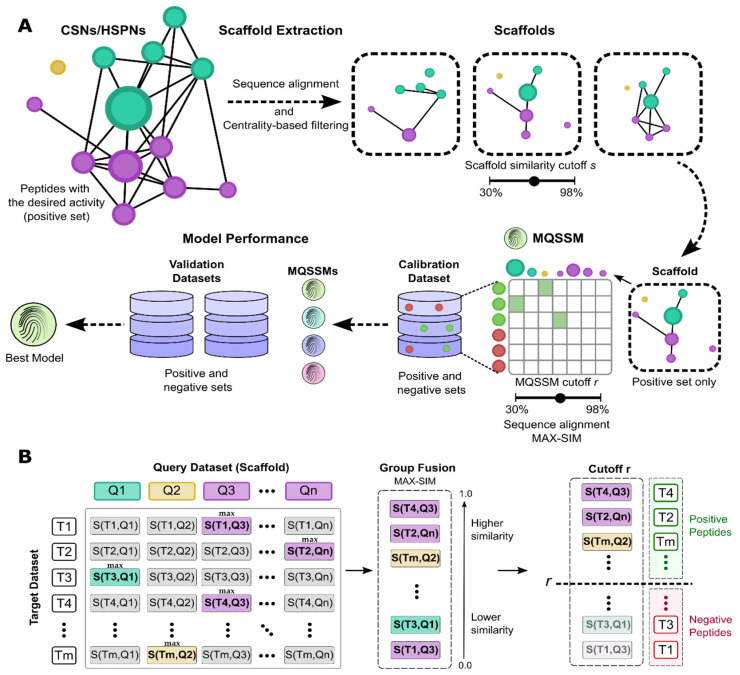
Process to develop model classifiers (MQSSMs) from informative peptide space networks. (**A**) Informative CSNs/HSPNs containing only peptides with a desired bioactivity are used to extract subsets of sequences, scaffolds, that take into consideration the centrality of each peptide in the network during the filtering process carried out using alignment-based pairwise comparisons. These scaffolds are the core of the MQSSMs which can identify peptides with the desired bioactivity from a dataset of peptides. MQSSMs are first calibrated using a dataset with known positive and negative sequences, then the similarity cutoff of the model is fine-tuned until the model reaches a high performance. Subsequently, these models can be further improved by fusing different scaffolds or by enriching them with sequences excluded from the initial network. Finally, the performance of the models is assessed in external datasets. (**B**) Description of the multi-query similarity searching method. A pairwise similarity measure, $S(T_{i},Q_{j})$, is calculated between each of the peptides in the query dataset (Q) and each of the peptides in the target dataset (T). Local and global alignments can be used to calculate the similarity between peptides. Then, for each target peptide, the MAX-SIM rule is defined as follows: $max\{S\left(T_{i},Q_{j})\right\};\forall T_{i}\in T$ is applied (Group Fusion). The resulting similarity values are ranked from the most to the least similar. Finally, a similarity cutoff value *r* is selected, so that it maximizes the accuracy and performance of the model to discriminate positive from negative peptides. Target peptides with higher similarity values than *r* are considered as positive samples. This figure was adapted from ref. [42].

MQSSMs represent a topology-informed alternative to conventional supervised prediction approaches. Rather than learning decision boundaries from labeled training sets, MQSSMs rely on the selection of representative peptides (scaffolds) from complex networks (CSNs or HSPNs) and use them as a positive class of multiple similarity queries against target peptides (Figure 6A). This makes MQSSMs fundamentally different from standard supervised classifiers, which depend on the explicit partition of data into positive and negative classes and are often affected by biases in training set composition [25,75,76].

A critical step in MQSSM construction is the extraction of peptide scaffolds from network topology; this process is guided by the results of community detection and centrality analysis. Specifically, scaffolds are selected from network communities by prioritizing peptides occupying informative topological positions, including highly central nodes as well as atypical nodes such as singletons [33,44]. The rationale is that central peptides capture the dominant structural features of a community, whereas atypical nodes may preserve peripheral or singular regions of the chemical space that would otherwise remain underrepresented.

Several representative scaffolds are extracted from a network using different centrality measures. Then, peptides are ranked according to their centrality and pairwise comparisons are conducted between peptides. If two peptides have a sequence similarity (global or local) higher than a cutoff (s), the one with lower centrality is removed. The remaining peptides constitute a scaffold. This process of redundancy reduction prevents the scaffold from being dominated by multiple variants of essentially the same peptide pattern [6,34].

### 6.2. Multi-Query Search and Model Refinement

MQSSMs perform prediction by using the selected scaffold as a set of multiple queries against a target calibration database with positive and negative classes. The key methodological principle at this stage is group fusion, whereby the similarity outputs produced by multiple queries are combined to generate a final prediction for each candidate peptide. This is implemented through the MAX-SIM rule, in which the maximum similarity score obtained between a target peptide from the calibration dataset and any of the query peptides in the scaffold is used as the final decision value (Figure 6B) [33,44].

The use of MAX-SIM is conceptually simple but powerful. It assumes that a candidate peptide may resemble one relevant region of the active chemical space even if it is not globally similar to all query scaffolds. By retaining the strongest match across the scaffold set, MQSSMs allow the model to capture heterogeneous manifestations of a given biological function while avoiding the restrictive assumptions of single-query searches or rigid class boundaries.

Selection of a similarity cutoff (r) for an MQSSM is essential as this establishes the threshold at which peptides from the target dataset are considered similar to the positive set of peptides represented by the scaffold. This cutoff is fine-tuned using the calibration dataset by assessing the performance of the model that maximizes the discrimination between positive and negative classes. MQSSMs can be further improved by fusing peptides from different scaffolds that have been obtained using different centrality measures, cutoffs (s), or even from networks created using different (dis)similarity metrics [42]. Furthermore, bioactive peptides from outside StarPepDB can be incorporated into the workflow to enrich sequence representativeness. Finally, the best-performing models are then tested in external validation datasets that do not have peptides in common with StarPepDB.

MQSSMs have been shown to achieve performance comparable to, and often exceeding, that of machine learning–based predictors in tasks such as the classification of antiparasitic [33], tumor-homing [44], hemolytic [42], and antiviral peptides [25]. These results are central to the significance of the framework because they show that network-derived, similarity-based predictive systems can achieve strong empirical performance without relying on standard supervised training pipelines.

A major strength of MQSSMs is their interpretability. Unlike many supervised models, which may function as black-box classifiers [77], MQSSMs preserve an explicit connection between prediction and peptide space organization. The selection of scaffolds is topologically grounded, the similarity rule is transparent, and the final decision can be traced back to one or more representative reference peptides. Furthermore, the framework is less dependent on the availability of large, labeled training sets and is therefore particularly attractive in problems where class definitions are incomplete or where reliable negative sets are difficult to establish [78].

## 7. Representative Implementations of Graph-Based Peptide Informatics

The methodological advances described in the previous sections require computational resources capable of operationalizing graph-based data integration, descriptor-based peptide representation, network construction, topological analysis, and similarity-based exploration. In this section, StarPepDB [23], StarPep Toolbox [34], and StarPepWeb [12] are discussed as representative implementations of the graph-based peptide informatics framework reviewed here, with emphasis on how these resources illustrate the progressive translation of complex network concepts into usable, accessible, and reusable computational environments.

**Figure 7:**
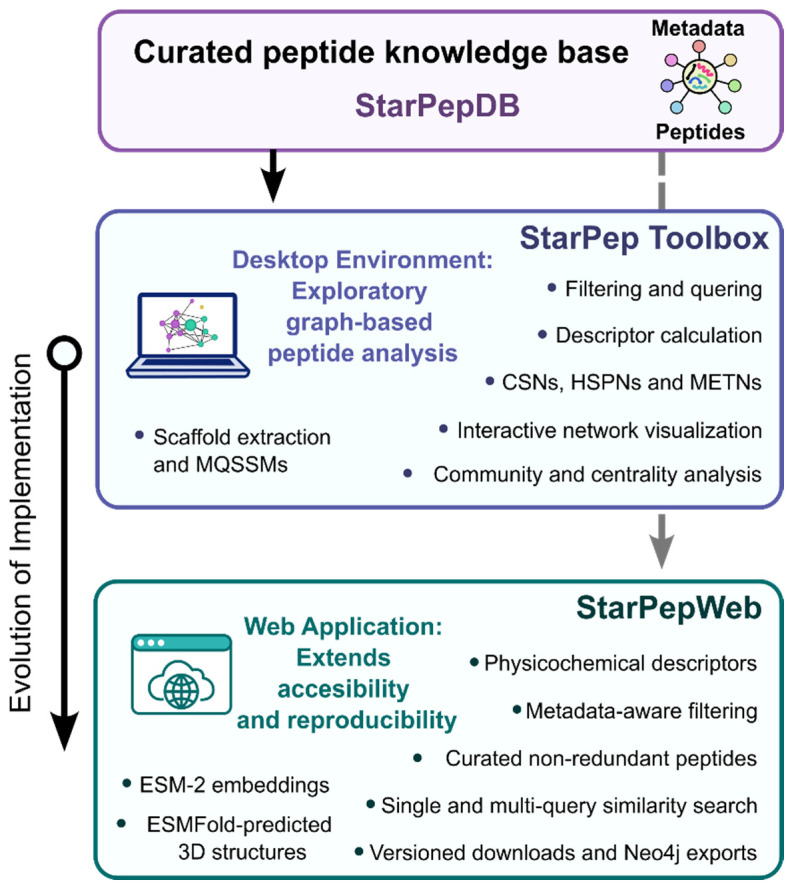
Representative implementation layers of graph-based peptide informatics. StarPepDB provides the curated peptide knowledge base, integrating peptide sequences and metadata within a graph-based structure. StarPep Toolbox represents the desktop analytical layer, enabling filtering and querying, descriptor calculation, construction of CSNs, HSPNs, and METNs, interactive network visualization, community and centrality analysis, scaffold extraction, and MQSSM development. StarPepWeb broadens access to StarPepDB through a web-accessible and reproducible environment, providing enriched peptide data, physicochemical descriptors, ESM-2 embeddings, ESMFold-predicted structures, metadata-aware filtering, single- and multi-query search, versioned downloads, and Neo4j exports. Together, these resources illustrate the progression from curated graph-based data integration to standalone visual analytics and web-accessible reuse of enriched peptide knowledge.

In graph-based peptide informatics, implementation is not a secondary aspect of the framework, but a critical component that determines how curated peptide data, descriptor-based representations, network construction, and topology-driven analyses can be explored, interpreted, and reused by researchers. In this context, the progression from StarPepDB [23] to StarPep Toolbox [34] and StarPepWeb [12] reflects the maturation of the field from graph-based data integration and standalone visual analytics toward web-accessible, extensible, and reproducible peptide informatics infrastructures (Figure 7).

### 7.1. Desktop Implementation: StarPep Toolbox as a Visual Analytics Environment

StarPep Toolbox was developed as an open-source desktop application for exploring peptide chemical space through graph-based analysis and visual data mining. Its implementation integrated several core components of the methodological framework reviewed here, including peptide filtering and querying, optimized molecular descriptor calculation, construction of metadata and similarity networks, and development of multi-query similarity-searching models. Rather than functioning only as a visualization utility, the toolbox served as an operational layer connecting curated peptide resources with network science and similarity-based workflows. The StarPep Toolbox software and its installation files are freely available at the project GitHub repository (https://github.com/Grupo-Medicina-Molecular-y-Traslacional/StarPep, accessed on 5 May 2026).

A defining strength of StarPep Toolbox is its support for interactive exploration of chemical space representations and network topology. The software allows users to inspect communities, central nodes, singletons, scaffold candidates, and shortest paths; customize layouts and node attributes; and visually examine how peptide organization changes across network constructions. In this sense, the toolbox operationalizes the principles of visual information seeking in graph-based peptide informatics, allowing researchers to move beyond static summaries toward interactive, interpretable, and topology-aware analyses of peptide organization [34].

### 7.2. Web-Accessible Implementation: StarPepWeb and the Enriched StarPepDB Resource

StarPepWeb should be understood primarily as a freely accessible web application that extends and democratizes access to StarPepDB (https://mobiosd-hub.com/starpep/, accessed on 5 May 2026) [23], rather than as a direct web analog of the standalone visual analytics toolbox. The platform harmonizes entries from 40 public peptide databases into a curated, non-redundant, source-tracked graph resource containing 45,120 bioactive peptides enriched with metadata, physicochemical features, ESMFold-predicted 3D structures, ESM-2 embeddings, and iFeature-derived descriptors. In this sense, StarPepWeb functions as an integrative web resource for peptide retrieval, filtering, similarity search, and reproducible data access. StarPepWeb is freely available through its public web interface, whereas the source code and documentation are hosted at its dedicated GitHub repository (https://github.com/starpep-web, accessed on 5 May 2026).

Its methodological significance lies in making this enriched version of StarPepDB broadly accessible through a browser-based interface organized around search, statistics, downloads, and source-code access. The web platform supports metadata-aware filtering, alignment-based single- and multi-query searches, interactive visualization, and versioned bulk downloads, including Neo4j exports for offline graph mining. Its modular, microservice-oriented architecture enables scalable deployment, maintainability, and reproducible updates, while its data-centric design prioritizes uniform peptide representations and versioned reusable exports, leaving predictive analyses to downstream offline workflows rather than embedding them directly in the platform [12].

### 7.3. Toward Scalable and Reproducible Peptide Informatics Infrastructures

Taken together, StarPep Toolbox [34] and StarPepWeb [12] represent complementary implementation layers within graph-based peptide informatics (Figure 7). StarPep Toolbox operationalizes the analytical and visual exploration of peptide chemical space, whereas StarPepWeb expands access to the underlying curated graph resource through a web-accessible, extensible, and versioned platform. This progression reflects the transition of the field from specialized analytical software toward reusable computational infrastructures that support large-scale peptide retrieval, standardized representation, and reproducible downstream analysis.

## 8. Conclusions and Future Perspectives

Graph-based peptide informatics has evolved into a coherent framework for exploring the chemical space of bioactive peptides. Its value lies not only in visualization, but in the integration of curated repositories, descriptor-based representations, similarity and metadata networks, topological analyses, and topology-informed predictive strategies within a unified and interpretable analytical paradigm.

Nevertheless, these approaches also have methodological limitations that should be considered. Network results depend on descriptor choice and feature selection, while CSNs are particularly sensitive to threshold selection. In addition, database bias, redundancy, incomplete annotations, and inconsistent metadata can affect downstream interpretation. Scalability may also become challenging for very large peptide collections, especially when dense pairwise similarity matrices or highly connected networks are generated. These limitations highlight the need for transparent descriptor selection, careful threshold analysis, curated data sources, and scalable implementations.

A major strength of this framework is that it complements conventional supervised learning, especially when negative datasets are scarce, class boundaries are diffuse, and peptide functional space is heterogeneous. By exploiting the intrinsic organization of similarity space, these approaches convert network topology, node centrality, community structure, and scaffold representativeness into useful analytical features for peptide characterization, prioritization, and prediction.

The field has also progressed from conceptual models to practical infrastructures. The transition from StarPepDB to StarPep Toolbox, and to StarPepWeb reflects the consolidation of peptide network science into accessible, extensible, and reproducible computational platforms for large-scale peptide retrieval, exploration, and reuse.

Future developments will likely depend on integrating richer sequence, structural, and metadata representations into scalable environments while preserving methodological transparency, reproducibility, and principled evaluation. In this context, hybrid strategies that combine interpretable network abstractions with modern representation learning appear especially promising. Overall, graph-based approaches now provide a structured, interpretable, and computationally tractable paradigm for peptide analysis, discovery, and design.

## Data Availability

No new data were created or analyzed in this study. Data sharing is not applicable to this article.
